# Enhancement of Micropollutant Degradation at the Outlet of Small Wastewater Treatment Plants

**DOI:** 10.1371/journal.pone.0058864

**Published:** 2013-03-06

**Authors:** Luca Rossi, Pierre Queloz, Alessandro Brovelli, Jonas Margot, D. A. Barry

**Affiliations:** Institut d'ingénierie de l'environnement, Faculté de l'environnement naturel, architectural et construit, Ecole polytechnique fédérale de Lausanne, Lausanne, Switzerland; National University of Singapore, Singapore

## Abstract

The aim of this work was to evaluate low-cost and easy-to-operate engineering solutions that can be added as a polishing step to small wastewater treatment plants to reduce the micropollutant load to water bodies. The proposed design combines a sand filter/constructed wetland with additional and more advanced treatment technologies (UV degradation, enhanced adsorption to the solid phase, e.g., an engineered substrate) to increase the elimination of recalcitrant compounds. The removal of five micropollutants with different physico-chemical characteristics (three pharmaceuticals: diclofenac, carbamazepine, sulfamethoxazole, one pesticide: mecoprop, and one corrosion inhibitor: benzotriazole) was studied to evaluate the feasibility of the proposed system. Separate batch experiments were conducted to assess the removal efficiency of UV degradation and adsorption. The efficiency of each individual process was substance-specific. No process was effective on all the compounds tested, although elimination rates over 80% using light expanded clay aggregate (an engineered material) were observed. A laboratory-scale flow-through setup was used to evaluate interactions when removal processes were combined. Four of the studied compounds were partially eliminated, with poor removal of the fifth (benzotriazole). The energy requirements for a field-scale installation were estimated to be the same order of magnitude as those of ozonation and powdered activated carbon treatments.

## Introduction

Micropollutants such as pharmaceuticals, personal care products and biocides are ubiquitous in the environment [1]–[3]. Due to poor removal in conventional wastewater treatment plants (WWTPs) [4]–[5], urban areas are among the major sources of micropollutants. They are often biologically active at low concentrations (ng l^−1^ to µg l^−1^), and have diverse deleterious effects on aquatic organisms and ecosystems [6]–[15]. This problem has been increasingly recognized in recent years, resulting in new measures to improve their removal. In Switzerland, for example, implementation of novel treatment solutions to reduce the micropollutant load could soon become mandatory for WWTPs serving areas with more than 8,000 population equivalent [16].

Two technologies have proved effective for micropollutant removal: ozonation and adsorption onto powdered activated carbon [4]–[5], [17]. Although effective, advanced treatment technologies have high construction and maintenance costs, high energy consumption and require qualified permanent staff for their operation (ozonation in particular), making their implementation feasible only for medium/large scale WWTPs [18]. On the other hand, small WWTPs (<5000 population equivalent, 54% of 867 WWTPs in Switzerland) treating wastewater from villages and farms release smaller amounts of micropollutants, which can nevertheless degrade the quality of the receiving water bodies [19]. Low-cost and low-maintenance alternative treatment solutions that can reduce micropollutant concentrations are therefore of continuing interest for small WWTPs.

Constructed wetlands (CWs) are an efficient and cost-effective alternative to traditional WWTPs in many situations. They have long been used for the treatment of urban wastewater at small scale and to attenuate diffuse contamination of surface waters due to agricultural runoff [20]–[22]. Although mainly used to remove organic carbon, suspended solids and nutrients (e.g., [23]), CWs have shown their potential to remove recalcitrant compounds [2], [24]–[34]. Their effectiveness varies from negligible to total depending on the physico-chemical characteristics of the micropollutants, wastewater composition, properties of the CWs, and environmental conditions (e.g., temperature) [35]. For many compounds, the removal efficiency is the same or even better than that observed in conventional WWTPs [5], [36]–[37]. Still, highly recalcitrant compounds such as carbamazepine or clofibric acid with limited or negligible removal have been noted [36], [38]–[39].

To ensure adequate elimination of most, if not all, contaminants, conventional processes such as biodegradation are insufficient. Here, we complement classical CWs with additional treatment steps, termed the Engineered Constructed Wetland (ECW) approach. The aim of this work is to outline the main characteristics of such systems, and to present the results of a preliminary study conducted to evaluate ECW feasibility.

An ECW is a subsurface flow CW divided into cells or compartments. Each cell is designed to host or sustain a specific treatment process, and can either be filled with a porous material or equipped with a treatment technology. To keep maintenance, energy requirements and running costs low, most cells will host a passive (i.e. conventional) treatment system, using either a natural (e.g., sand, peat, etc.) or engineered (e.g., light expanded clays, iron-coated sand, granular activated carbon) substrate. Some compartments can instead be equipped with more advanced treatment technologies (such as UV) to guarantee a satisfactory elimination rate of a specific class of compounds. Artificial aeration can also be considered to homogenize the water column and to promote oxic conditions that are more favorable for micropollutant biodegradation [40]. Sustainability of such a system is enhanced by incorporation of passive power supply (e.g., solar).

ECW technology is modular and flexible, and therefore improvements to treat specific compounds or accommodate changes in specifications are easily implemented. Apart from the advantages inherent to CWs, the key strength of the ECW approach is that it exploits the synergy between natural and engineered processes. For example, in a typical setup, upstream passive treatments involving a planted sandy substrate filters the wastewater, thereby reducing the turbidity and removing a large fraction of the more labile contaminants through biodegradation. An open compartment with UV light then transforms photodegradable recalcitrant compounds. Downstream of the UV compartment, passive systems involving biodegradation and adsorption remove possible toxic by-products of the photodegradation. An approach similar to the ECW concept, using different units of processes in a phytoremediation wetland, was already proposed [41]. However, the treatment processes were not focused directly on micropollutants.

In this work, a preliminary evaluation of the ECW approach is presented using five common micropollutants with different physico-chemical characteristics and different levels of environmental persistence. The behavior of these micropollutants is investigated first in batch containers, and then a in laboratory scale flow-through system that combines different advanced treatments. The goals are to evaluate the degree to which these pollutants are eliminated and to determine to what extent insights obtained in batch experiments (which are abundant in the literature) can be used to infer micropollutant behavior in flow-through systems.

## Materials and Methods

### Micropollutant selection and analysis

The compounds used in this study (Table 1) were selected considering the indicators included in the proposed revision of Switzerland's water protection law [16]: carbamazepine (CBZ), an anticonvulsant and mood-stabilizing drug, diclofenac (DCF), a non-steroidal anti-inflammatory and analgesic drug and sulfamethoxazole (SMX), a sulfonamide antibiotic drug. Benzotriazole (BZT), a corrosion inhibitor widely used in industrial processes, dishwashing agents and deicing fluids and mecoprop (MCP), a common herbicide used in many household weed killers, green roof sealing protection and lawn fertilizers, were also studied. For these compounds, WWTPs should guarantee an elimination rate of at least 80%. They are all recalcitrant to biodegradation [5], only partially degraded in WWTPs and can be persistent in the environment. A summary of their ecotoxicity and chemical-physical properties is presented in Table 1.

**Table 1:** Summary of physico-chemical and ecotoxicological properties of the substances considered in this study, and information on the analytical procedures and experiments.

	Diclofenac	Carbamazepine	Mecoprop	Benzotriazole	Sulfamethoxazole
CAS no	15307-86-5	298-46-4	93-65-2	95-14-7	723-46-6
Use	Anti-inflammatory	Anticonvulsant	Herbicide	Corrosion inhibitor	Antibiotic
log K_ow_ a	4.02	2.25	2.99	1.23	0.89
pKa b	4.18	13.94	3.19	8.38	5.81
EQS c (ng l^−1^)	50	500	1000	30,000	600
LOD/LOQ (ng l^−1^)	1.6/3.7	0.5/2	4/12	2/5	2/6
SPE-LC/MS/MS recovery rate (%)	77–101	95–102	74–115	62–97	79–109
Inlet concentrations (µg l^−1^) d	0.3/1.5	0.5/0.5	1/2.5	−/120	−/0.14

a [94]–[96].

b Calculated from ACD/Labs (www.acdlabs.com, last accessed 19 January 2013).

c Environmental Quality Standards [97]–[100].

d First/second experiment.

The micropollutants were purchased from Sigma-Aldrich (Buchs, Switzerland). Internal standards used for analytical validation were purchased from CDN Isotopes (Quebec, Canada) for carbamazepine-d10 and from Toronto Research Chemicals (Toronto, Canada) for diclofenac-d4 and benzotriazol-d4. The micropollutant solutions were prepared in ultrapure water from an individual stock solution prepared in methanol, and stored at −20°C until use in amber bottles to avoid photodegradation.

Samples were analyzed by first adjusting them to pH 2 with hydrochloric acid, followed by solid phase extraction (SPE, GX-274 ASPEC; Gilson). Three-cc OASIS HLB cartridges (60 mg sorbent, Waters®) were used for the experiments. Cartridges were conditioned with 6 ml of methanol followed by 6 ml of deionized water (pH 2) at a flow rate of 1 ml min^−1^. A total of 500 ml of each sample was extracted at 10 ml min^−1^. After drying cartridges for 20 min, compounds were eluted with 6 ml of methanol at 1 ml min^−1^. After extraction, the samples were further concentrated in high-grade methanol using a nitrogen stream to obtain a final volume of 0.5 ml. The quantification was performed using an Ultra Performance Liquid Chromatograph (UPLC) coupled to tandem quadrupole mass spectrometers (Acquity TQD from Waters®). Details of instrumentation and MS/MS parameters are given elsewhere [42]. Samples were spiked with the internal standards Diclofenac-d4 and Benzotriazole-d4 before SPE, and with Carbamazepine-d10 before the analysis with UPLC-MS/MS to compensate for the matrix effect and to determine recovery rates. For the batch adsorption tests, only the internal standard 10,11-dihydrocarbamazepine was added in prior to SPE. The detection/quantification limits are provided in Table 1. Our laboratory regularly participates in national and international laboratory ring tests to ensure the quality of the analyses [43].

### Experimental design

Table 2 reports a summary of the batch experiments conducted to assess the ability of individual processes to remove micropollutants. For all compounds, the UV degradation kinetics were studied. Regarding sorption, three media (sand, Filtralite® and LECA) were initially considered, but tests with sand and Filtralite® were conducted only on CBZ. The quartz sand had particle size between 0.5 and 1.6 mm and a bulk density of about 1500 kg m^3^ (Carlo Bernasconi AG, Switzerland). Filtralite® is an expanded clay commercial product with high porosity and surface area. The chemical and physical characteristics of the beads make it suitable for many applications, included use as a filter material for constructed wetlands. LECA (Light Expanded Clay Aggregate) is conventionally used in agriculture, and has been shown to adsorb micropollutants [5], [16]–[18]. As discussed further in the results section, the silica sand used was found to have a negligible sorption capacity. Filtralite® induced satisfactory removal if properly proportioned, but with a significant pH increase (>10 for fresh Filtralite®) due to calcium hydroxide release [44]. These findings are consistent with previous studies (e.g., [44]–[45]), and led to the conclusion that both sand and Filtralite® are not suitable substrates. For this reason, they were not used in subsequent experiments.

**Table 2:** Summary of batch experiments conducted to study the removal capacity of individual treatments. All experiments were repeated three times.

	Sorption			UV degradation
	Sand	Filtralite®	LECA	
CBZ	X	X	X	X
DCF	-	-	X	X
MCP	-	-	X	X
SMX	-	-	X	X
BZT	-	-	X	X

Batch experiments were limited to a short period (24 h) since short residence times are inherent in the ECW concept (ECW installations would have a limited size in practice).

Batch experiments were complemented with measurements in flow-through systems to investigate pollutant removal with coupled treatment processes and to quantify the effect of reduced mixing (in contrast to well-stirred batch experiments). Two similar flow-through setups were used. In one, the substrate was silica sand, while in the other LECA was added. The two experiments were designed to evaluate the relative importance of adsorption versus UV degradation.

### Batch tests

Standardized OECD laboratory procedures for batch adsorption experiments were followed [46]. The support material was dried first in air and then for 24 h in an oven at 107°C (sand and Filtralite®) or at 120°C (LECA) to eliminate water and sterilize the samples. The filter materials were maintained in a desiccator to avoid humidification prior to weighing.

#### Sorption of CBZ on sand and Filtralite®

For the batch adsorption tests with sand and Filtralite®, a 2-μg l^−1^ CBZ solution was prepared with successive dilutions to allow precise weighing. A volume of 250 ml of the final solution was added to glass columns (Ø 6 cm, 700 ml) containing different amounts of porous substrate, in order to obtain three soil-to-solution ratios (1/1, 1/5 and 1/25). A control sample (CBZ solution only) and blanks (filter material with ultrapure water only) were prepared also. The columns were shaken for 22 h at ambient temperature (20–25°C). The solution was extracted from the solid matrix by 20-min centrifugation at 7000 rpm and filtered with 0.45-μm mixed cellulose ester membrane filters (Whatman). The samples were prepared and analyzed in duplicates.

#### Sorption of DCF, MCP, CBZ, SMX and BZT on LECA

A solution with 1.5 μg l^−1^ DCF, 2.5 μg l^−1^ MCP, 0.5 μg l^−1^ CBZ, 0.14 μg l^−1^ SMX and 120 μg l^−1^ BZT was prepared with ultrapure water for the batch adsorption tests with LECA. Glass columns were filled with LECA/solution at a ratio of 1/1.8. Blank samples (without LECA) were tested to evaluate possible adsorption on the apparatus. The columns were shaken for 24 h at ambient temperature. The liquid phase was collected after filtration using 0.45-μm filters (Whatman). Samples were prepared and analyzed in triplicate.

#### Photodegradation of DCF, MCP, CBZ, SMX and BZT

To evaluate photodegradation rates, batch experiments were conducted with UV-C light using a solution of micropollutants with the same concentrations as the LECA adsorption batch tests. A 700-ml laboratory reactor with a 15-W low-pressure mercury UV lamp from Heraeus Noblelight (Germany) was used (emission at 254 nm). The micropollutant solution (500 ml) was exposed to UV under constant stirring. Four exposure times (1, 3, 10, 30 min) were used to characterize the reaction kinetics. The exposure times used in the experiments were determined assuming first-order degradation rates [47] and preliminary screening tests. The solution was adjusted to pH 7.5 prior to exposure to correspond to the typical pH of WWTP effluent.

### Combined treatments in a flow-through setup

**Figure 1:**
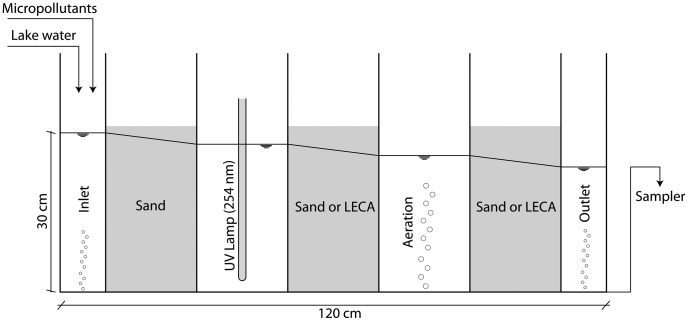
Schematic illustration of the flow-through experiment, similar to an ECW system but unplanted. This setup was used for the experiments in this work, and therefore the dimensions reported in the drawing are not representative of a full-scale system.

A sketch of the laboratory flow-through system is shown in Figure 1. This system is intended to mimic the behavior of advanced treatments in an ECW.

The laboratory-scale model was made of transparent Plexiglas and consisted of five treatment compartments, plus the inlet/outlet cells. The first, third and fifth compartments were filled with a porous substrate for adsorption and filtration (sand or LECA), whereas the second compartment was equipped with a UV lamp (of the same type used in the batch tests). The fourth compartment was equipped with an aeration system. The cells were separated by Plexiglas-perforated walls with uniformly-spaced 10-mm holes (16 in 100 cm^2^) and a removable plastic mesh to maintain the porous medium in place. The inflow rate was regulated by a volumetric flow meter (Vögtlin V100-140.11, Aesch, Switzerland). The outlet was connected to a flexible pipe that allowed adjustment of the hydraulic head. Water from Lake Geneva (Switzerland) stored in a large reservoir at constant temperature (20°C) was supplied continuously. The micropollutant solution (stored in a continuously stirred 20-l amber bottle) was added in the inlet cell by a peristaltic pump. The inlet compartment was mixed continuously using an aquarium bubbler. A similar device was added to the outlet cell to avoid possible stratification due to density differences (e.g., [48]).

The outlet pipe was connected to a continuously stirred 500-ml Erlenmeyer. Samples were collected in this receptacle using an automatic sampler (Teledyne ISCO 6712, Lincoln, USA, with a 24-PE bottle kit).

Micropollutant degradation was tested using two different setups. In the first setup, the first, third and fifth compartments were filled with quartz sand (particle sizes between 0.5 and 1.6 mm, porosity of 0.32, Carlo Bernasconi AG, Switzerland). In the second setup, the third and fifth compartments were filled with LECA (porosity of 0.46). Some weeks before the experiments, the system was inoculated with treated wastewater from the Lausanne WWTP to foster the development of acclimatized microbial consortia.

To compare the results of the two cases, the same mean hydraulic residence time (HRT, 6 h) was used. LECA had a higher hydraulic conductivity than sand, and therefore the hydraulic head difference was adjusted (285 and 270 mm for the first and second experiments, respectively), giving a flow rate of 2.8 l h^−1^, corresponding to a hydraulic loading rate of 2240 mm d^−1^. Each experiment was divided into two phases: injection (3 d) and washout (4 d). During the injection phase, a solution of micropollutants was added at a flow rate of 0.2 l h^−1^ with a peristaltic pump and diluted in the inlet basket with a lake water inflow of 2.6 l h^−1^. After mixing with lake water, the micropollutant concentrations at the inlet were 0.3 μg l^−1^ of DCF, 0.5 μg l^−1^ of CBZ and 1 μg l^−1^ of MCP for the first experiment and 1.5 μg l^−1^ of DCF, 0.5 μg l^−1^ of CBZ, 2.5 μg l^−1^ of MCP, 120 μg l^−1^ of BZT and 0.14 μg l^−1^ of SMX for the second. Concentrations of micropollutants were in the range of measured substances at the outlet of real WWTPs in Switzerland [49]. Sodium chloride (1.5 g l^−1^ in the inflow) was used as a tracer to quantify the hydraulic behavior of the system. This concentration of NaCl is not expected to interact with the different compounds. The electrical conductivity (EC) was measured at the inlet and outlet with Hach CDC401 IntelliCAL probes (Düsseldorf, Germany) every 15 min. Water samples of 500 ml each were collected from the system effluent every 3 h during the injection phase and every 6 h during washout. Samples were filtered and acidified (the maximum interval between sampling and filtration was 15 h) before being extracted following the analytical procedure described previously.

### Energy requirements

Unlike gravity-driven classical CWs, ECWs require external energy. The estimation of the amount of energy needed for the UV process and oxygenation was based on similar studies and literature review. The results were compared with energy requirements estimated for classical advanced treatment for micropollutant elimination in the same socio-economical context, like ozonation and adsorption onto powdered activated carbon [5].

## Results and Discussion

### Batch adsorption tests

**Figure 2:**
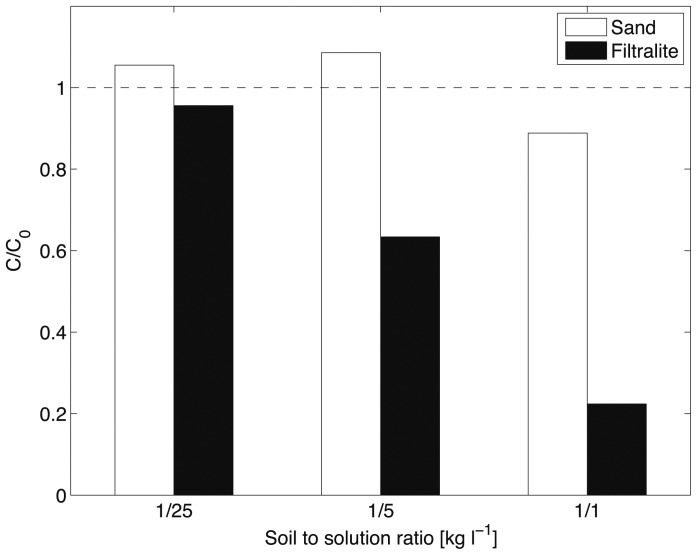
Relative concentration of CBZ after adsorption onto sand and Filtralite® in batch tests. Normalized concentrations larger than unity are likely due to analytical uncertainties.

The analysis of the blank samples for the batch adsorption tests on sand and Filtralite® for CBZ showed little adsorption to the vessel. The measured concentration of both replicates was above 97% of the initial concentration. The mean concentration of the blank samples was taken as the initial concentration to calculate the degradation rate of the other samples. The results shown in Figure 2 reveal poor CBZ adsorption (11%) onto sand with the larger soil-to-solution ratio (1/1), while with smaller ratios no removal was observed. This small removal confirms the poor adsorption capacity of clean sand.

**Figure 3:**
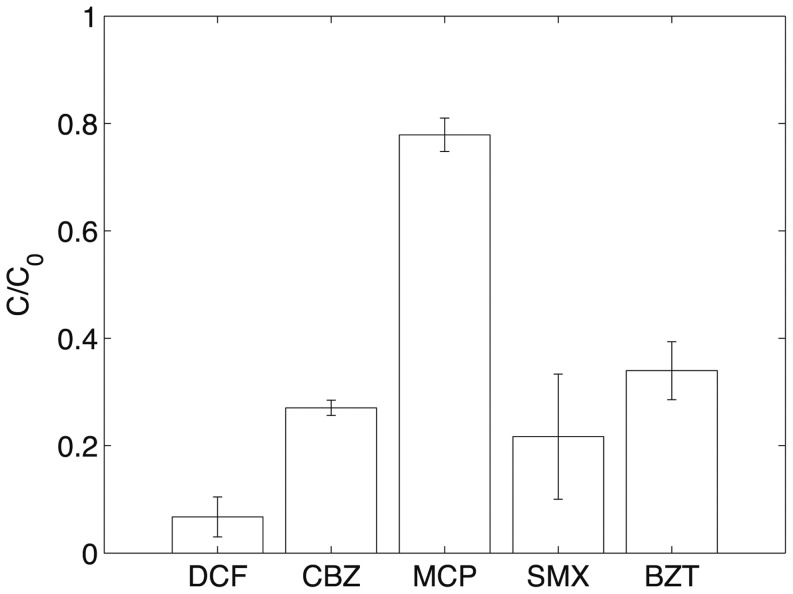
Relative concentration ±1 standard deviation of DCF, CBZ, MCP, SMX and BZT after adsorption onto LECA in batch tests. The LECA-to-solution ratio in these experiments was 1/1.88 kg l^−1^. Except for MCP, the micropollutants have medium-to-high affinity for LECA.

In contrast, Filtralite® efficiently removed the CBZ if proportioned at a sufficient soil-to-solution ratio. With 0.2 kg l^−1^, 37% of CBZ in the solution was removed, and up to 78% with a ratio of 1 kg l^−1^. These findings are consistent with previous observations [27]. CBZ is mildly hydrophobic and remains un-dissociated at neutral pH (see log K_ow_ and pK_a_, Table 1). Due to the presence of silanol groups, the silica surface is hydrophilic and negatively charged. The low affinity of the hydrophobic molecules considered in this study (e.g., CBZ) for silica is therefore not unexpected and explains the negligible sorption of most micropollutants. On the contrary, lightweight aggregates such as Filtralite® have a large specific surface area, which facilitates adsorption even if the affinity for the organic molecules is only moderate (the inner surface of Filtralite® and other LECAs is typically positively charged, see, e.g., [50]). This is confirmed in Figure 3, which shows the results of the batch adsorption tests on LECA for the five substances. Adsorption of CBZ on LECA was higher than the removal using Filtralite®. The initial concentration was reduced by 73% after 24 h at a soil-to-solution ratio of about 0.5 kg l^−1^. LECA has a similar adsorption capacity for SMX and BZT with, respectively, 78% and 66% removal. DCF was adsorbed efficiently by LECA (93% removal). In contrast, LECA only partially removed MCP (26% removal was observed).

The results of the adsorption experiments are in agreement with theoretical results and previous reports (Table 3). In particular, different studies suggested that electrostatic interactions control SMX adsorption onto mineral surfaces at neutral pH values [51]–[52]. BZT and SMX have similar hydrophobicity, and a similar removal in the adsorption experiments was observed. The significant sorption of DCF can be explained by its negative charge, suggesting electrostatic attraction with the positively charged LECA surface at neutral pH. Among the studied compounds, only MCP showed low sorption on LECA. This result cannot be explained by the charge and the hydrophobicity of the molecule alone, which are very similar to those of the other compounds. Previous work on MCP fate in soils and other porous materials (e.g., [53]–[54]) reached a similar conclusion. For example, low adsorption and consequently high mobility of MCP in a variety of soils (from organic to calcareous) over the pH range 7.2–8 was reported [55]. In addition, lower adsorption of MCP on activated carbon in a full scale wastewater treatment plant was measured [5]. Overall, our results confirm those of Dordio et al. [50], who showed the influence of different media on the removal of micropollutants in CWs. Based on our results and those in the literature (e.g., [56]–[60]), LECA has potential as a suitable adsorption medium but it requires further investigation of its adsorption properties for the target compounds, especially its long-term performance. Moreover, adsorption of micropollutants in a complex matrix like wastewater is expected to be lower due to the competition for adsorption sites [60].

**Table 3:** Summary of the degradation efficiency for the five selected micropollutants in different wastewater treatment systems.

Type of processes and specifications			Parameters				
			DCF	CBZ	MCP	BZT	SMX
Classical CW processes	Adsorption a	Sand/Gravel	−	−	−−	−	−
		LECA	+	+	−−	+	+
	Biodegradation b	Anoxic	−−	n.a	−−	n.a	n.a
		Aerobic	+	−	+	+	n.a
	Plant uptake c	*P australis* or *Typha* sp.	−	++	−−	n.a	n.a
Advanced processes	Direct photolysis d	Near UV (200–400nm)	++	−	+	+	+
	Advanced oxidation processes e	UV (185 nm)	++	+/−	n.a	n.a	+
		UV (254 nm)/H_2_O_2_	++	+	n.a	n.a	++
		O_3_	++	++	+	++	+
CWs	In/out measurements f	Subsurface CWs	−	−	−	n.a	+
WWTPs	Activated sludge systems g	Without nitrification	−	−	−	−	+/−
		With full nitrification	−	−	+/−	−	−
Classical CWs	Sand only		−−	−−	−	n.a	n.a
ECW	Sand		++	+/−	+	n.a	n.a
	LECA		++	+/−	+	−−	++

Removal >95%: ++, 70–95%: +, 30–70%: +/−, 5–30%: –, <5%: – –, not available: n.a. Upper part of the table refers to literature review, the lower part (grey area) to this study.

a [25], [31], [38], [40], [101]–[105].

b [36], [63], [106]–[109].

c [38]–[39], [110].

d [47], [61], [111].

e [47], [62], [64], [111]–[115].

f [25], [27]–[28], [33], [36], [38], [103], [116], [117].

g [5].

### UV photodegradation batch tests

**Figure 4:**
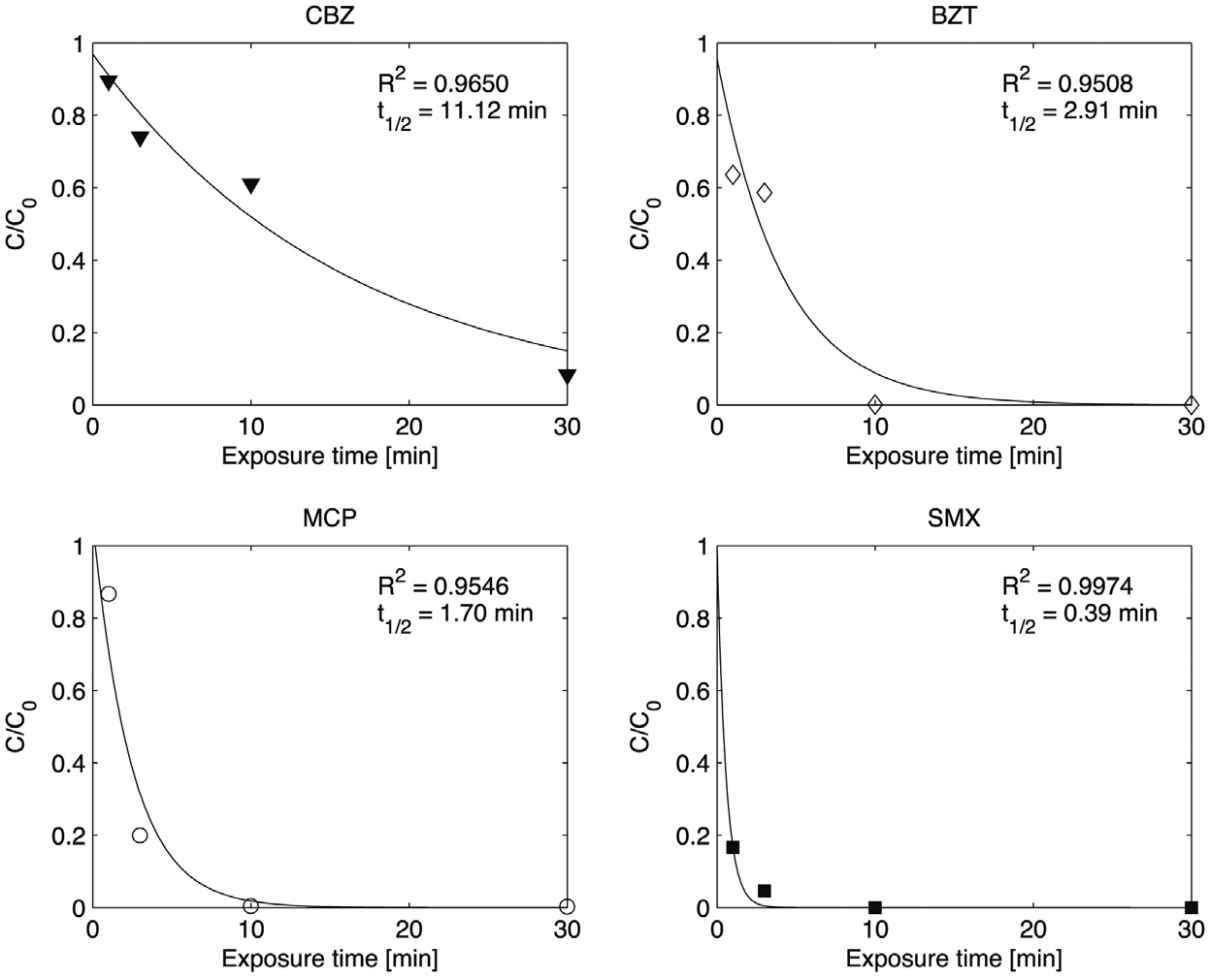
UV degradation kinetics at pH 7.5 for CBZ, BZT, MCP and SMX. The solid line shows a fitted first-order exponential decay. The correlation coefficient (R^2^) for the fit and the expected half-life (t_1/2_) are also reported.

The UV degradation tests were conducted at pH 7.5 since urban WWTP effluents have typically nearly neutral pH. In Figure 4, degradation kinetics are reported, except for DCF. This substance is easily photodegradable (removal was completed in less than 1 min), with concentrations all below the analytical detection limit. The solid line in each plot is an exponential model fitted to the measurements, constrained by *C*(t = 0)/*C*_0_ = 1, to estimate the first-order degradation rate and half-life. DCF, MCP and SMX decayed rapidly, with negligible residual concentrations after 10 min. BZT was also efficiently photodegraded in the first 10 min, although at a slightly lower rate. CBZ is the most recalcitrant compound, as the normalized residual concentration after 30 min was about 15%.

The results of the experiments show that photolysis can lead to high removal rates with a relatively short residence time. This conclusion is in agreement with previous reports, as shown in Table 3 ([61]–[64]). Kim and Tanaka [47] studied photolysis of DCF, SMX and CBZ at concentrations in the μg l^−1^ range using an 8-W low pressure mercury lamp emitting light at 254 nm, at pH 7. DCF and SMX were classified as easily photodegradable substances with first-order rate constants greater than 2.6×10^−3^ s^−1^ (i.e., 90% degradation in less than 15 min). CBZ instead was classified as moderately degradable, with a first-order rate constant of 6.4×10^−4^ s^−1^ giving 90% degradation within 1 h. The degradation of BZT with concentrations in the mg l^−1^ range (i.e., at concentrations much higher than those usually found in treated wastewaters) was explored with a medium to high-pressure lamp [62] at pH 7 and 8. At pH 7, around 20% to almost 90% reduction of BZT was achieved for UV doses from 34 to 1070 mJ cm^−2^. At pH 8, approximately 20% reduction was achieved regardless the intensity of the UV source. Note that these results were obtained with a higher UV energy than used here. Photolysis of BZT in aqueous solutions was reported by Liu et al. [65]. In that study, the degradation rate was much lower (half-life was around 2.3 h) than measured in our study (about 5 min), but with different experimental conditions. The importance of the effect of pH on the photolytic degradation of BZT was also reported by Andreozzi et al. [66]. Lower pH values enhance the degradation kinetics of compounds, but do not reflect the usual pH values measured at WWTP outlets. Adsorption materials that increase wastewater pH (e.g., Filtralite®) would therefore reduce the efficiency of further UV-degradation [44].

Photodegradation usually creates several photoproducts that could be more toxic than the parent compound, e.g., DCF [67]. Thus, further treatment is necessary to remove the potentially toxic photoproducts after the UV treatment. This could be achieved by adsorption or biodegradation in a further compartment of the ECW. For instance, the effluent toxicity diminished after treatment in an ozonation-sand filter system, relative to ozonation alone [68]–[69]. The intensity of UV light (500 mJ cm^−2^) was much higher than that conventionally used for water disinfection at the WWTP outlet (120–160 mJ cm^−2^, [70]–[71]). A positive side effect of UV is water disinfection.

### Flow-through experiments

**Figure 5:**
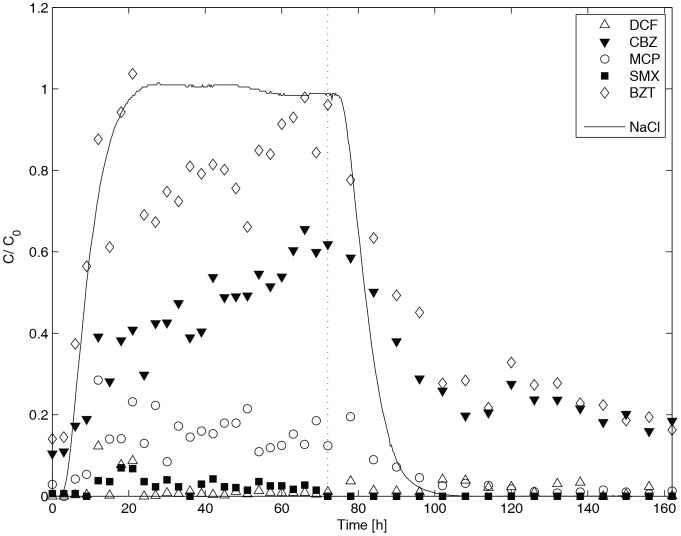
Relative degradation of DCF, CBZ, MCP, SMX, BZT in the laboratory-scale ECW with LECA and sand as adsorption support, together with UV photolysis. The vertical dashed line corresponds to the end of the injection phase.

The results of the laboratory-scale experiments with combined treatments are illustrated in Figure 5, where effluent concentrations from the injection and washout phases are presented. In general, the micropollutant removal efficiencies are consistent with the results of adsorption and UV photolysis batch tests.

DCF and SMX are both well degraded, with the effluent relative concentration always below 10%. These two substances were easily photo-degraded by UV, and strongly adsorbed onto LECA. DCF is poorly adsorbed to sand [36] and LECA (results not reported), indicating that UV photolysis was the key degradation process.

MCP was also removed efficiently (degradation above 80%) by UV photolysis. This compound also has low adsorption onto LECA (Figure 3) and sand. The change over time of CBZ can be attributed to sorption onto LECA (possibly also to mass transfer into the stagnant water phase inside the aggregates).

Similar behavior was found for BZT. This is due to sorption/desorption onto LECA, as suggested again by the batch experiments. On the other hand, the results indicate reversible sorption, i.e., BZT is not permanently retained on the surface. One possibility is that the observed retardation is controlled by the mass transfer between mobile and immobile porosity (i.e., from the bulk water to inside the aggregates). In this case, the residence time in the compartments filled with LECA would not be sufficient to achieve irreversible sorption.

Results for BZT are also not in agreement with UV photodegradation batch tests. Low removal was observed at the end of the injection step, despite high affinity of this compound for LECA and a relatively rapid degradation rate in the batch UV tests. The residence time in the LECA compartments was under 24 h, and so adsorption equilibrium was probably not reached, leading to lower adsorption. Moreover, photochemical degradation of BZT is strongly pH-dependent. Two forms of BZT are possible depending on the pH (non-ionic in acidic conditions and ionic in basic conditions), with each form having different photoreactive behavior. The ionic form is much less reactive than the un-dissociated molecule [72], where it was shown that the UV degradation evolves from exponential decay at pH 7 to roughly linear decay at pH 9. Photolysis of BZT in the flow-through system is comparable to the investigation of Liu et al. [65]. In contrast, in a real, vertical flow CW with stabilized biological degradation processes, 93% BZT elimination was reported [28]. For a horizontal CW with a large residence time (720 h), temperature was identified as important factor for BZT degradation (degradation during winter months: 0%, during summer months: 53%). From this comparison, it appears that detailed understanding of the mechanisms and physico-chemical variables that control UV degradation of BZT are still unclear [73].

Overall, the somewhat contradictory results obtained in the flow-through system in comparison with the batch experiments illustrate the challenge of combining different processes. Clearly, our results show that the overall CW performance is not as simple as summing the results of the individual processes.

### Micropollutants in the context of treatment wetlands

Table 3 summarizes the different individual processes that occur in a CW. These are compared with engineered processes (direct photolysis, advanced oxidation, ozonation), degradation of selected micropollutants observed in classical subsurface flow CWs (in-out measurements) and in WWTPs with or without full nitrification, as well as the results of the experiments conducted in this work.

Recall that Filtralite® was not used as an adsorption material in the ECW. Preliminary tests revealed the tendency of Filtralite® to increase the pH to a level incompatible with UV degradation. This highlights the difficulty of combining different processes that may have antagonistic or, in the best case, synergetic effects on ECW efficiency.

Experiments conducted to investigate the ECW concept, based on realistic micropollutant concentrations, showed promising results, enhancing the degradation of DCF, MCP and SMX. Removal efficiencies observed in the laboratory-scale experiments for these compounds were comparable with those obtained using advanced treatment processes in WWTPs [5]. A further increase of the degradation rates could be expected for an ECW in practice. In particular, the laboratory system had several limitations: (i) it was not planted, (ii) the residence time was relatively short (6 h) compared to realistic CWs, and (iii) the contribution of biodegradation was limited, probably due to lack of nutrients that limited biomass growth. Experiments were conducted with lake water, which contains limited amounts of carbon and nutrients. But even in this case, results showed promising results for future use of the ECW concept. Detailed modeling and experiments with real/synthetic wastewater would be necessary to extend our results for a real case application.

Experience with classical CWs shows that variability in removal rates is large, and is influenced by climatic (i.e., temperature) and design parameters, such as residence time and age of the installation [23], [74]. In addition, previous CW experiments [75] suggested that biodegradation played a more active role than in our laboratory setup, where photodegradation was significant. In fact, as is the case with activated sludge wastewater treatment, microbial degradation can play an important role in removing micropollutants such as pharmaceuticals and herbicides in treatment processes based on CWs [31]. However, little is known about the fate of such compounds and their metabolites in CWs. The higher efficiency of biodegradation in previous studies could be related to the higher nutrient content in the wastewater and to the presence of plants, which create environments suitable for biological activity in the rhizosphere, and the development of stable microbial communities able to degrade micropollutants by secondary substrate catabolism or cometabolism [31], [37], [76]–[78].

Artificial aeration of CWs has already been considered by different authors to promote, for example, nitrogen elimination [79], to enhance degradation processes during cold months [80] or for landfill leachate treatment [81]. More oxidized conditions promote also biochemical reactions that are beneficial for micropollutant degradation [38], [40], [82], [83]. The free-water bubbling system breaks down vertical heterogeneities in the water column linked with heterogeneous flow fields in the filtering material [74]. Biodegradation and adsorption can therefore be accentuated over the entire depth of the system.

Experiments on a laboratory-planted ECW or better on a full scale ECW will be necessary to investigate the role of biological degradation of micropollutants. Direct extrapolation of the flow-through results to a full scale ECW system is challenging due to possible scale effects. But, as already noted, our laboratory results are in agreement with those from larger scale experiments. In our case, the advanced treatment approach alone is already appropriate for eliminating the selected micropollutants. The combination of these processes with biodegradation, as found in classical CWs, could lead to a high degradation rate for the studied substances in ECWs.

### Energy requirements

ECWs require external energy sources, in particular for the UV treatment and aeration. For the UV system, a calculation of the energy needs was conducted based on information from [84] for a dedicated UV source (Heraeus Noblelight^TM^, 30W UV lamp, 80-cm height). With a water transmittance of 90% and 98% elimination of DCF (UV dose of 500 mJ cm^−2^), the energy need was estimated at 0.21 kWh m^−3^. A study on UV degradation of SMX with UV/H_2_O_2_ coupled processes yielded an estimate of 0.11 kWh m^−3^ for 90% degradation [85]. Note that, in this last case, lake water was considered, thus substantially less energy was required due to lower scavenging rates compared with treated wastewater. However, UV-LED technology is evolving rapidly and could contribute in the near future to reducing the energy consumption [86].

This energy calculation for micropollutant elimination is based on UV only, so the presence of other elimination processes (adsorption, biodegradation) in the ECW will enhance the capacity of the system to eliminate these substances and therefore energy needs would be lower. UV degradation efficiency depends on different factors, like contact time, turbidity, pH and the characteristics of UV radiation [71]. The UV degradation module could be located near the end of the ECW, in order to eliminate pollutants that have not been degraded by the previous steps. Alternatively, it could be positioned at the beginning of the ECW, as in our case, to eliminate recalcitrant compounds and to favor degradation of metabolites. As already mentioned, a biologically active filtration step after the UV treatment cell would likely be necessary to eliminate potential ecotoxic metabolites. Due to the preliminary filtration processes, water turbidity is in fact minimized. For example, water transmittance after sand filtration in the system used in this work was estimated at 98%. Contact time depends on the residence time of the water in the system, and can normally be adjusted during operations.

For the aeration system, the energy demand is also dependent on parameters like the oxygen transfer rate, initial and final dissolved oxygen concentrations, temperature and water depth. In the setup we are considering, the goal is to avoid anaerobic zones and to ensure good mixing of the water column. As a first approximation, energy requirements were estimated at 0.06–0.08 kWh m^−3^ of treated water considering an airflow rate five times greater than water flow rate [87].

Different sustainable energy sources of energy are possible: photovoltaic, wind, hydraulic with different storage and conversion modes: autonomous, semi-autonomous and grid-connected. For example, for Swiss conditions (city of Lausanne), the best solution found was photovoltaic panels with a grid-connected system [88]. Wind turbines or micro-hydraulic energy are possible also, but depend on local conditions [89]–[92].

By comparison, energy requirements for advanced treatments for the elimination of micropollutants at the outlet of a WWTP were estimated at 0.11 kWh m^−3^ for ozonation followed by sand filtration, and between 0.095 and 0.9 kWh m^−3^ for adsorption onto powdered activated carbon (PAC) followed by either sand filtration/UV or ultrafiltration [5]. Note that in this latter case the UV step achieved water disinfection. The energy needs for UV treatment and aeration in the ECW are still high, but the maintenance costs associated with ozonation or PAC technologies do not appear. There is a potential to decrease this energy requirement by optimization of the processes or using UV-LED technology.

Our experiments indicate the potential of the ECW concept. However, accurate design of the setup is critical to achieve high elimination efficiency and reliability of the system. For this task, detailed process-based modeling of micropollutants is needed. To date, however, most CW models concentrated on classical parameters, such as carbon load and macronutrients (nitrogen and phosphorous primarily, e.g., [93]). Only few modeling attempts have been conducted on compounds that can be classified as micropollutants (e.g., [74]).

## Conclusions

Micropollutants represent an important challenge for water treatment. For example, Swiss authorities are expected to impose discharge requirements for indicative substances. Even though advanced solutions have been tested successfully on large WWTPs, the needs of small WWTPs remain, especially when the discharged waters end up in small streams. Constructed wetland systems are potential solutions for the removal of micropollutants. However, large differences in their elimination rates highlight the need of better understanding of degradation processes, and particularly the need for including more advanced elimination processes to ensure water quality. It is likely that use of ECWs as a polishing step for small WWTPs will also have positive effects on removal of other pollutants, enhancing their degradation and eliminating residual nutrient concentrations, retaining TSS (total suspended solids) and adsorbed compounds and allowing water disinfection. Therefore, the ECW approach could play a useful role in the concept of water reuse, as a sustainable approach to water management.

The ECW paradigm represents an innovative adaptation of traditional CWs, coupling natural processes and advanced treatment technologies. Based on our preliminary results and the potential identified in different processes, elimination of micropollutants in line with the 80% elimination target of anticipated future Swiss legislation seems to be achievable. The final concentrations in our experiments were all below the EQS (Environmental Quality Standard) threshold. Nevertheless, information is still needed on the different processes prior to dimensioning a real system for micropollutant elimination. For this, the next step will be to build a real ECW that includes also mature biodegradation processes and plants.

The investigation of selected degradation processes so far highlights the importance of the wetland substrate, and the beneficial input of UV in an ECW design approach. The compartmented approach allows for optimization of degradation processes in individual cells. In addition it has potentially a positive synergetic effect, allowing for example optimization of UV degradation after filtration. Research is still needed for the optimization of the ECW, especially in sizing the system for the characteristics of the water to be treated, in optimizing the energy consumption and in improving our understanding of the degradation processes. This will lead to the development of an optimal sustainable advanced water treatment system for small communities.
